# Lésions coronaires chez le noir africain dans les syndromes coronariens aigus

**DOI:** 10.11604/pamj.2019.32.104.12637

**Published:** 2019-03-05

**Authors:** Hermann Yao, Arnaud Ekou, Thierry Joseph Niamkey, Esaïe Kossa Soya, Emilienne Aboley, Roland N'Guetta

**Affiliations:** 1 Service des Soins Intensifs Médicaux, Institut de Cardiologie d'Abidjan, Abidjan, Côte d'Ivoire; 2 Service de Médecine, Institut de Cardiologie d'Abidjan, Abidjan, Côte d'Ivoire; 3 Service des Explorations Fonctionnelles, Institut de Cardiologie d'Abidjan, Abidjan, Côte d'Ivoire

**Keywords:** Syndrome coronarien aigu, coronarographie, Afrique subsaharienne, Acute coronary syndrome, coronary angiography, Sub-Saharan Africa

## Abstract

**Introduction:**

les syndromes coronariens aigus (SCA) sont en progression en Afrique sub-saharienne. Les données angio-coronarographiques sont rares. L'objectif de cette étude était d'analyser les lésions coronaires observées dans les syndromes coronariens aigus (SCA) à Abidjan.

**Méthodes:**

étude transversale, du 1^er^ janvier 2010 au 31 décembre 2014. Tous les patients admis pour SCA et ayant bénéficié d'une coronarographie à l'Institut de cardiologie d'Abidjan pendant la période d'étude ont été inclus. Deux cent cinquante-six patients ont été sélectionnés. Nous avons analysé et comparé les lésions coronaires chez les patients ayant présenté un SCA avec sus décalage persistant du segment ST (SCA ST+) et ceux ayant présenté SCA sans sus décalage du segment ST (SCA ST-).

**Résultats:**

l'âge moyen des patients était de 53,2 ± 10,8 ans. Il existait une prédominance masculine avec un sex-ratio de 6,1. Le taux de coronarographies anormales était significativement plus élevé dans les SCA ST+ (95,4% et 64,2% respectivement, p < 0,001). Trois cent quatre lésions coronaires ont été observées dans les SCA ST+ contre 43 dans les SCA ST- . Les lésions sténosantes étaient majoritairement monotronculaires (45,3%) dans les SCA ST+ et bi ou tri tronculaires dans les SCA ST- (68,0%). Les lésions de type B1 étaient plus fréquentes dans les SCA ST- (62,8% versus 36,5%, p = 0,002). Les lésions les plus complexes de type C étaient plus fréquentes dans les SCA ST+ (17,8%), sans différence significative. La majorité des patients avait un score SYNTAX inférieur à 22 quel que soit le type de SCA (87,4% dans les SCA ST+ et 90,1% dans les SCA ST-).

**Conclusion:**

les lésions coronaires dans les SCA sont majoritairement monotronculaires dans les SCA ST+ et plus diffuses dans les SCA ST- mais avec une proportion plus importante de coronaires angiographiquement normales. La prise en charge de ces lésions relève dans la majorité des cas de l'angioplastie coronaire.

## Abstract (en)

**Introduction:**

acute coronary syndromes (ACSs) have been increasing in sub-Saharan Africa. Coronary angiography data have been rarely reported. This study aims to investigate coronary lesions observed in patients with acute coronary syndromes (ACS) in Abidjan.

**Methods:**

we conducted a cross-sectional study from 1^st^ January 2010 to 31^st^ December 2014. All patients with ACS admitted to the Abidjan Heart Institute and undergoing coronary angiography during the study period were included. Two hundred and fifty-six patients were selected. We investigated and compared coronary lesions in patients with ACS associated with persistent ST-segment elevation (ACS ST+) and in those with ACS without ST-segment elevation (ACS ST-).

**Results:**

the average age of patients was 53.2 ± 10.8 years; there was a male predominance, with a sex ratio of 6.1. Abnormal coronary angiography rate was significantly higher in the STEMI group (95.4% and 64.2% respectively, p < 0.001). Three hundred and four coronary lesions were found in the STEMI group against 43 in the NSTE-ACS group. Stenotic lesions were predominantly one-vessel disease (45.3%) in the STEMI group and two or three-vessel disease in the NSTE-ACS group (68.0%). Type B1 lesions were more common in the NSTE-ACS group (62.8% versus 36.5%, p = 0.002). The most complex type C lesions were more commonly found in the STEMI group (17.8%), with no significant difference. The majority of patients had SYNTAX score less than 22, whatever the type of ACS (87.4% in the STEMI group and 90.1% in the NSTE-ACS group).

**Conclusion:**

coronary lesions in the patients group with STEMI were predominantly one-vessel disease and they were more diffuse in the NSTE-ACS group, but with a higher proportion of angiographically normal coronary arteries. The management of patients with these lesions is based in the majority of cases on coronary angioplasty.

## Introduction

Les pays en voie de développement, particulièrement ceux d'Afrique subsaharienne, connaissent ces dernières décennies une transition épidémiologique, marquée par l'expansion des maladies cardiovasculaires, et particulièrement des coronaropathies [1-4]. Si les particularités épidémiologiques et cliniques des syndromes coronariens aigus (SCA), mode d'expression le plus grave de la maladie coronaire, ont été décrites, très peu de séries angiographiques ont été rapportées [5]. En outre, le traitement percutané des lésions coronaires, notamment dans les SCA est désormais possible dans certains pays d'Afrique subsaharienne [3, 4, 6, 7]. L'objectif de ce travail était d'analyser les lésions coronaires dans les SCA dans une population Afrique subsaharienne, afin de mieux organiser leur prise en charge.

## Méthodes

Notre étude a eu pour cadre le service d'hémodynamique et de cardiologie interventionnelle de l'Institut de Cardiologie d'Abidjan (ICA), centre national de référence pour la prise en charge des affections cardiovasculaires en Côte d'Ivoire. Nous avons mené une étude transversale sur une période de 4 ans, du 01^er^ janvier 2010 au 31 décembre 2014. Ont été inclus de façon consécutive tous les patients âgés d'au moins 18 ans, admis à l'ICA pour SCA et ayant bénéficié d'une coronarographie pendant la période d'étude, après qu’ils aient donné leur consentement éclairé. N'ont pas été inclus dans l'étude, les patients ayant présenté une contre-indication ou ayant refusé l'examen et ceux dont la coronarographie n'avait pas été réalisée à l'ICA. Les paramètres étudiés étaient: 1) Les données sociodémographiques et anthropométriques: l'âge (en années), le genre (masculin ou féminin), le poids (kg), la taille (cm), l'index de masse corporelle (kg/m²), le tour de taille (cm). 2) Les antécédents et facteurs de risque de maladie coronaire: l'hypertension artérielle, le tabagisme, le diabète sucré, les dyslipidémies, l'obésité, l'histoire familiale de maladie coronaire ou d'accident vasculaire cérébral, l'antécédent de syndrome coronarien aigu, d'angioplastie coronaire ou de pontage coronarien. 3) La présentation clinique: les patients étaient répartis en deux grands groupes selon les anomalies électrocardiographiques et l'élévation ou non des marqueurs biologiques de nécrose myocardique: les SCA avec sus-décalage persistant du segment ST (SCA ST+), les SCA sans sus décalage persistant du segment ST à Troponine I positive (SCA ST- T+) ou négative (SCA ST-T-). 4) L'échographie cardiaque: les troubles de la cinétique segmentaire (akinésie, hypokinésie ou dyskinésie), la fonction systolique du ventricule gauche. 5) la coronarographie: elle permettait de faire le bilan lésionnel coronaire. La coronarographie était considérée comme normale lorsque les artères coronaires étaient lisses, sans plaque d'athérome, sans phénomènes spastiques. L'athérome non sténosant a été décrit lorsqu'il existait une réduction inférieure à 70% du calibre de référence sur les artères épicardiques et inférieur à 50% sur le tronc commun. La sténose est un rétrécissement de la lumière d'un vaisseau supérieur ou égal à 70% et supérieur ou égal à 50% pour le tronc commun avec présence d'un flux en aval. L'occlusion est une oblitération totale de la lumière avec une absence de flux en aval [8]. En présence d'une maladie coronaire obstructive (sténose ou obstruction), les lésions étaient classées selon le nombre de vaisseaux atteints (monotronculaire, bitronculaire, tritronculaire) et selon leur sévérité (classification de l'ACC/AHA [9] et score SYNTAX [10]). 6) La prise en charge: la revascularisation coronaire (par angioplastie ou pontage aorto-coronarien), les médications associées. Les données ont été saisies et analysées à l'aide du logiciel Epi Data 3.1. L'analyse statistique a été réalisée avec le logiciel Epi info 7. Les variables catégorielles, présentées sous forme d'effectifs et de pourcentages, ont été comparées par le test de Chi-carré ou le test exact de Fisher. Les variables continues, présentées sous la forme de moyenne ± écart type (m ± ET) ont été comparées par le test de Student. Le seuil de significativité a été retenu pour une valeur de p < 0,05.

## Résultats

Sur 580 patients ayant présenté un SCA de 2010 à 2014, 256 ont pu bénéficier d'une coronarographie, soit un taux de réalisation de la coronarographie de 44,1%. Les caractéristiques générales de la population d'étude sont résumées dans les Tableau 1, Tableau 2, Tableau 3. L'âge moyen était de 53,2 ± 10,8 avec des extrêmes de 26 et 78 ans. Les tranches d'âge les plus touchées étaient comprises entre 51 et 60 ans (36,7%) ainsi que la tranche d'âge comprise entre 41 et 50 ans (24,2%). Il existait une nette prédominance masculine (sex-ratio=6,1). La prise en charge de la coronarographie était aux frais des patients dans 74,2% des cas. Seul un quart des patients bénéficiaient d'une couverture maladie. La présentation clinique était dominée par le SCA ST+ (68,4%). La coronarographie était réalisée dans les 24 heures chez 22,7% des patients. Le taux de coronarographies anormales était significativement plus élevé dans les SCA ST+ (p < 0,001). Un tiers des lésions siégeait sur l'artère interventriculaire antérieure (IVA) dans les deux présentations cliniques soit 35,5% (108/304) pour le SCA ST+ et 32,5% (14/43) pour le SCA ST-. Les lésions monotronculaires étaient plus fréquentes dans les SCA ST+ (45,3%), sans différence significative au plan statistique. Dans les SCA ST-, les lésions étaient plus souvent bi ou tritronculaires dans 68% des cas, de façon non significative. Les lésions de type B1 étaient significativement plus fréquentes dans les SCA ST-. Les lésions les plus complexes de type C étaient plus fréquentes dans les SCA ST+ sans différence significative. La majorité des patients avait un score SYNTAX inférieur à 22, prédisant un bon pronostic pour l'angioplastie. Cent dix patients (43,0%) ont bénéficié d'une angioplastie coronaire, majoritairement dans les SCA ST+. L'implantation d'une endoprothèse a été réalisée dans la très grande majorité des cas (91,8%). Le pontage aorto-coronarien a été réalisé chez 3 patients présentant un SCA ST-.

**Tableau 1:** caractéristiques générales de la population d’étude

Caractéristiques générales	SCA ST+ (n=175)	SCA ST- (n=81)	p
***FDRCV et antécédents***			
Age (années), m±ET	53,6 ± 10,9	52,3 ± 10,8	0,37
Sexe masculin	155(88,6)	65(80,2)	0,07
IMC (kg/m²), m±ET	26,4 ± 3,8	27,7 ± 3,9	0,06
Hypertension artérielle	89(50,9)	50(61,7)	0,10
Diabète	33(18,9)	19(23,5)	0,39
Tabagisme actif	52(29,7)	17(21,0)	0,14
Hypercholestérolémie	46(26,3)	18(22,2)	0,48
Obésité	35(20,0)	22(27,2)	0,20
Hérédité coronarienne	13(7,4)	3(3,7)	0,40
Syndrome coronarien aigu	26(14,9)	13(16,0)	0,80
Antécédents d’angioplastie	9(5,1)	4(4,9)	0,81
***Délai symptômes-admission***			
< 6 heures	35(20,0)	4(4,9)	0,001
6-12 heures	18(10,3)	5(6,2)	0,28
13-24 heures	19(10,8)	5(6,2)	0,23
>24 heures	103(58,9)	67(82,7)	<0,001
***Territoire électrocardiographique***			
Antérieur	83(47,4)	13(16)	< 0,001
Inférieur	67(38,3)	38(46,9)	0,19
Latéral	15(8,6)	27(33,3)	< 0,001
Circonférentiel	10(5,7)	3(3,7)	0,76
***Echocardiographie***			
FEVG (%), m±ET	51,5 ± 13,7	63,9 ± 10,8	< 0,001
Troubles de la contractilité	109(62,3)	11(13,6)	< 0,001

Les données sont en n(%), autrement elles ont été précisées. m±ET: moyenne ± écart type. SCA ST+: syndromes coronariens aigus avec sus-décalage du segment ST. SCA ST-: syndromes coronariens aigus sans sus-décalage du segment ST. FDRCV: facteur de risque cardiovasculaire. IMC: indice de masse corporelle. FEVG: fraction d’éjection ventriculaire gauche.

**Tableau 2:** aspects angio-coronarographiques

Aspects coronarographiques	SCA ST+(n=175)	SCA ST-(n=81)	p
***Délai de réalisation de la coronarographie***			
0 -12 heures	17(9,7)	1(1,2)	0,01
13-24 heures	24(13,7)	16(19,8)	0,21
25-72 heures ****	21(12,0)	9(11,1)	0,83
>72 heures	113(64,6)	55(67,9)	0,60
***Dominance coronaire***			
Droit dominant	167(95,5)	70(86,4)	0,01
Gauche dominant	6(3,4)	10(12,3)	0,006
Equilibré	2(1,1)	1(1,1)	0,57
***Résultat de la coronarographie***			<0,001
Normal	8(4,6)	29(35,8)	
Anormal	167(95,4)	52(64,2)	
***Lésions sténosantes***	n=159	n=25	
Mono tronculaire	72(45,3)	8(32,0)	0,21
Bi tronculaire	61(38,4)	14(56,0)	0,09
Tri tronculaire	26(16,4)	3(12,0)	0,77
***Sévérité des lésions***	n=304	n=43	
Type A	62(20,4)	4(9,3)	0,12
Type B1	111(36,5)	27(62,8)	0,002
Type B2	77(25,3)	9(20,9)	0,53
Type C	54(17,8)	3(7,0)	0,11
***Score SYNTAX***			
0-22	153(87,4)	73(90,1)	0,53
23-32	12(6,8)	4(4,9)	0,75
≥ 33	10(5,7)	4(4,9)	0,96

Les données sont en n(%)

**Tableau 3:** distribution des patients selon les modalités de prise en charge

Modalités de prise en charge	SCA ST+ n=175	SCA ST-n=81	Total n=256
***Angioplastie***	95(54,3)	15(18,5)	110(43,0)
Ballon seul	8(8,4)	1(6,7)	9(8,2)
Stent nu	82(86,3)	12(80,0)	94(85,4)
Stent actif	5(5,3)	2(13,3)	7(6,4)
***Pontage aorto-coronarien***	0(0,0)	3(3,7)	3(1,2)
*Traitement médical seul*	80(45,7)	63(77,8)	143(55,8)

Les données sont en n(%)

## Discussion

Le taux de réalisation de la coronarographie était de 44,1% dans notre série. Ce taux est relativement faible comparativement aux résultats du registre français FAST-MI 2010 [11] qui retrouvait un taux de réalisation de la coronarographie de 96% et 91% respectivement chez les patients admis pour SCA ST+ et SCA ST-T+. Dans l'étude Euro Heart Survey-ACS II [12], la coronarographie était réalisée dans 70,2% des cas dans les SCA ST+, et chez 62,9% des patients au cours des SCA ST-. Ce faible taux de réalisation de la coronarographie s'explique par plusieurs facteurs, inhérents aux pays en développement. Le premier est représenté par les ressources humaines insuffisantes. La deuxième limite est liée au coût élevé de cette exploration: 400 000 FCFA (610 euros), soit 6,7 fois le salaire minimal interprofessionnel garanti en Côte d'Ivoire qui est de 60 000 FCFA (91 euros) [13]. Enfin, l'inexistence d'un système national d'assurance maladie universelle constitue un frein à la réalisation routinière de l'activité de cardiologie interventionnelle. Trois-quarts des patients de notre étude prenaient eux-mêmes en charge les frais de la coronarographie. Ce taux de réalisation dans notre contexte d'exercice reste cependant encourageant, dans un centre débutant comme le nôtre. Nos patients sont relativement jeunes, comme dans d'autres séries africaines [3, 14]. Dans les séries européennes et nord-américaines, les patients sont plus âgés, d'environ une décennie [11, 15]. Cette disparité pourrait s'expliquer d'une part par l'espérance de vie beaucoup plus élevée dans ces pays, mais aussi par l'absence de programmes efficaces de lutte contre les facteurs de risque cardiovasculaires en Afrique. Il existe une prédominance masculine dans notre série, conformément aux données de la littérature sur la maladie coronaire. Les facteurs de risque majeurs les plus fréquemment retrouvés étaient l'hypertension artérielle (54,3%), le tabagisme actif (27%), l'hypercholestérolémie (25%) et le diabète (20,3%). Ces facteurs étaient superposables à ceux retrouvée dans l'étude INTERHEART [14]. L'analyse de ces facteurs de risque ne montrait pas de différences significatives entre les présentations cliniques.

Les SCA ST+ (68%) étaient prépondérants dans notre série. Des résultats similaires étaient retrouvés au Kenya [4] et dans le registre FAST-MI 2010 [11]. A l'inverse, les SCA ST- étaient plus fréquemment rencontrés en Afrique du Sud [3] et dans le registre GRACE [16]. La majorité de nos patients (77,3%) bénéficiait d'une coronarographie au-delà de 24 heures. Cette observation est corrélée au long délai symptômes-admission retrouvé chez nos patients, qui était supérieur à 24 heures dans 66,4% des cas. Les moyens de transport utilisés par les patients, l'itinéraire précardiologique relativement long [2], et l'absence de structures préhospitalières spécialisées concourent à allonger les délais d'admission au niveau des services d'accueil des urgences. Les données angio-coronarographiques mettaient en évidence un taux de coronarographies anormales significativement plus élevé dans les SCA ST+ que dans les SCA ST- (95,4% et 64,2% respectivement, p < 0,001). Les lésions observées siégeaient majoritairement sur l'IVA quelque soit la présentation clinique, chez 35,5% des patients présentant un SCA ST+ et chez 32,4% des patients présentant un SCA ST- (p=0,70). La coronaire droite était concernée dans 29,3% des cas au cours des SCA ST+ et dans 25% des cas SCA ST- (p=0,11). Des données ivoiriennes antérieures [5] retrouvaient une atteinte préférentielle de l'IVA dans 43,1% des cas. Au Kenya, les résultats angiographiques dans les SCA ST+ étaient superposables: IVA (40%) et coronaire droite (33%). Les études réalisées sur les infarctus du myocarde ont décrit une répartition inégale des lésions, dont la majorité concerne l'IVA et la coronaire droite [17]. Le taux de coronarographies normales était significativement plus important dans les SCA ST- que dans les SCA ST+. La fréquence des SCA avec des artères coronaires normales rapportée dans la littérature varie de 8% à 12% [18-20]. Dans une étude réalisée en Espagne [18], 13% des patients atteints de SCA ST- n'avaient pas de sténose coronaire significative. Le sexe féminin, le jeune âge, l'absence de diabète et l'instauration d'un traitement antiplaquettaire au préalable étaient les facteurs associés à une absence de lésion significative à la coronarographie [18]. Dans le registre CRUSADE [21] l'absence de maladie coronaire obstructive a été retrouvée chez 10% des patients ayant bénéficié d'une coronarographie pour SCA ST-.

Plusieurs mécanismes ont été évoqués pour expliquer l'absence de lésion chez les patients ayant présenté un syndrome coronarien aigu: thrombus délité, dysfonction micro-vasculaire, spasme coronaire, embolie coronaire [22, 23]. Selon le nombre de troncs coronaires atteints, les données de notre étude retrouvaient 43,5% des patients monotronculaires, 40,8% bitronculaires et 15,8% tritronculaires. Dans les SCA ST+, les lésions monotronculaires étaient prédominantes. A Abidjan [5], un taux identique de lésions monotronculaires dans l'infarctus du myocarde (45,7%) avait été retrouvé. Dans les SCA ST-, l'atteinte coronaire était plutôt bi ou tritronculaire. Une observation similaire était rapportée au Kenya [4] où 56% des cas de SCA ST- présentaient des lésions coronaires bi ou tritronculaires. Nos observations sont similaires à celle de nombreuses études ayant rapporté les lésions coronaires observées dans les SCA [24, 25]. Concernant la sévérité des lésions coronaires, appréciée par la classification de l'ACC/AHA [9], les lésions de type B1 étaient significativement plus fréquentes dans les SCA ST-. Les lésions plus complexes de type C étaient plus fréquentes dans les SCA ST+. L'importance de ces lésions de type C traduit non seulement la gravité des lésions mais aussi les délais tardifs de prise en charge. En Tunisie, les lésions sévères de type B2 et C étaient fréquemment observées dans les SCA ST+ [26]. Dans notre série, une angioplastie coronaire a été réalisée chez 54,3% des patients admis pour un SCA ST+ et chez 18,5% des patients admis pour un SCA ST-. La majorité des patients avait un score SYNTAX inférieur à 22, ce qui prédisait un pronostic favorable pour l'angioplastie. Parmi les quatorze patients chez qui une indication opératoire était posée (SYNTAX score supérieur à 33), seuls trois patients ont pu être opérés. Aucun patient présentant un SCA ST+ n'a bénéficié de revascularisation chirurgicale.

## Conclusion

Les lésions observées dans les urgences coronaires sont variées. Elles sont importantes à préciser car elles déterminent en grande partie les modalités de prise en charge. Dans les SCA ST+, les lésions étaient majoritairement monotronculaires. Dans les SCA ST-, les lésions coronaires retrouvées étaient pluritronculaires, avec un taux plus important de coronaires angiographiquement normales. Les lésions observées dans les syndromes coronariens aigus dans notre pratique, restent dans une large mesure accessibles à l'angioplastie. D'où l'intérêt de promouvoir le développement de la cardiologie interventionnelle en Afrique subsaharienne.

### Etat des connaissances actuelles sur le sujet

- Dans les pays occidentaux, les lésions coronaires retrouvées sont majoritairement monotronculaires dans les syndromes coronariens aigus avec sus décalage du segment ST et pluritronculaires dans les syndromes coronariens aigus sans sus décalage du segment ST;
- Peu de données sont disponibles sur les lésions coronaires du sujets noir Africain après un syndrome coronaire aigu.

### Contribution de notre étude à la connaissance

- Les lésions coronaires retrouvées chez le Noir Africain, après un syndrome coronarien aigu, ont les mêmes caractéristiques que celles observées chez les Occidentaux;
- L'appréciation de la sévérité de l'atteinte coronaire au cours des syndromes coronariens aigus, grâce à des scores validés en Occident, a mis en évidence des lésions accessibles à un traitement percutané (angioplastie coronaire) avec de bons résultats prévisibles;
- La nécessité de la mise en œuvre et de la vulgarisation de la cardiologie interventionnelle en Afrique Subsaharienne, dans la prise en charge des syndromes coronariens aigus.

## References

[1] Touze JE (2007). Les maladies cardiovasculaires et la transition épidémiologique du monde tropical. Med Trop.

[2] N'Guetta R, Yao H, Ekou A, N'Cho-Mottoh MP, Angoran I, Tano M (2016). Prévalence et caractéristiques des syndromes coronariens aigus dans une population d'Afrique subsaharienne. Ann Cardiol Angéiol.

[3] Schamroth C, ACCESS South Africa investigators (2012). Management of acute coronary syndrome in South Africa: insights from the ACCESS (Acute Coronary Events-a Multinational Survey of Current Management Strategies) registry. Cardiovasc J Afr.

[4] Shavadia J, Yonga G, Otieno H (2012). A prospective review of acute coronary syndromes in an urban hospital in sub-Saharan Africa. Cardiovasc J Afr.

[5] Chauvet J, Renambot J, Ekra A, Ticolat R, Mouanodji M, Seka R (1991). Etude coronarographique et ventriculographique de 35 infarctus du myocarde chez des noirs africains à Abidjan. Cardiol Trop.

[6] N'Guetta R, Seka R, Ekou A, Anzouan Kacou JB, N'Cho-Mottoh MP, Adoh AM (2011). Percutaneous intervention in western Africa: experience of Ivory Coast. Cardiovasc J Afr.

[7] Johnson A, Falase B, Ajose I, Onabowale Y (2014). A Cross-sectional study of stand-alone Percutaneous Coronary Intervention in a Nigerian Cardiac Catheterization Laboratory. BMC Cardiovasc Disord.

[8] Winjs W, Kohl P, Danchin N, Di Mario C, Falk V, Folliguet T (2010). Guidelines on myocardial revascularization. Eur Heart J.

[9] Ryan TJ, Faxon DP, Gunnar RM, Kennedy JW, King SB, Loop FD (1988). Guidelines for percutaneous transluminal coronary angioplasty: a report of the American College of Cardiology/American Heart Association Task Force on Assessment of Diagnostic and Therapeutic Cardiovascular Procedures (Subcommittee on Percutaneous Transluminal Coronary Angioplasty). Circulation.

[10] Tajik P, Oude Rengerink K, Mol BW, Bossuyt PM (2013). SYNTAX score II. Lancet.

[11] Belle L, Cayla G, Cottin Y, Coste P, Khalife K, Labèque JN (2017). French Registry on Acute ST-elevation Myocardial Infarction (FAST-MI 2015): design and baseline data. Arch Cardiovasc Dis.

[12] Mandelzweig L, Battler A, Boyko V, Bueno H, Danchin N, Filippatos G (2006). The second Euro Heart Survey on acute coronary syndromes: characteristics, treatment and outcome of patients with ACS in Europe and the Mediterranean Basin in 2004. Eur Heart J.

[13] Côte d'Ivoire: le SMIG passe de 36.607 à 60.000 FCFA.

[14] Steyn K, Sliwa K, Hawken S, Commerford P, Onen C, Damasceno A (2005). Risk factors associated with myocardial infarction in Africa: the INTERHEART Africa study. Circulation.

[15] Peterson ED, Roe MT, Chen AY (2010). The NCDR ACTION Registry-GWTG: transforming contemporary acute myocardial infarction clinical care. Heart.

[16] Steg PG, Goldberg RJ, Gore JM, Fox KA, Eagle KA, Flather MD (2002). Baseline characteristics, management practices, and in-hospital outcomes of patients hospitalized with acute coronary syndromes in the Global Registry of Acute Coronary Events (GRACE). Am J Cardiol.

[17] Wang JC, Normand ST, Mauri L (2004). Coronary artery spatial distribution of acute myocardial infarction occlusions. Circulation.

[18] Cortell A, Sanchis J, Bodí V, Núñez J, Mainar L, Pellicer M (2009). Non-ST-Elevation Acute Myocardial Infarction With Normal Coronary Arteries: Predictors and Prognosis. Rev Esp Cardiol.

[19] Humphries KH, Pu A, Gao M, Carere RG, Pilote L (2008). Angina with “normal” coronary arteries: sex differences in outcomes. Am Heart J.

[20] Bugiardini R, Manfrini O, de Ferrari GM (2006). Unanswered questions in management of acute coronary syndrome. Arch Intern Med.

[21] Gehrie ER, Reynolds HR, Chen AY, Neelon BH, Roe MT, Gilber WB (2009). Characterization and outcomes of women and men with non-ST-segment elevation myocardial infarction and non obstrutive coronary artery disease: results from the Can Rapid Risk Stratification of unstable Angina patients Surppress Adverse Outcomes With Early Implementation of the ACC/AHA Guidelines (CRUSADE) quality improvement initiative. Am Heart J.

[22] N'Guetta R, Yao H, Ekou A, Boka B, Angoran I, Konin C (2015). Thrombose coronaire due à un déficit en protéine C: à propos d'un cas à Abidjan. Le Journal Africain du Thorax et des Vaisseaux.

[23] Ekou A, Yao H, N'Guetta R, Angoran I, Gnaba A, Koffi F (2015). Infarctus du myocarde à coronaires saines chez un sujet jeune après consommation de marijuana. Rev Int Sc Méd.

[24] Knot J, Kala P, Rokyta R, Stasek J, Kuzmanov B, Hlinomaz O (2012). Comparison of outcomes in ST-segment depression and ST-segment elevation myocardial infarction patients treated with emergency PCI: data from a multicentre registry. Cardiovasc J Afr.

[25] Arroyo Úcar E, Domínguez-Rodríguez A, Juárez Prera R, Blanco Palacios G, Hernández García C, Carrillo-Pérez Tome M (2011). Differential Characteristics of Patients with Acute Coronary Syndrome without ST-Segment Elevation Compared to those with Transient ST-Segment Elevation. Med Intensiva.

[26] Ben Ahmed H, Bouzouita K, Hamdi I, Ben Hassan F, Mokaddem A, Ben Ameur Y (2013). Caractéristiques angiographiques des septuagénaires hospitalisés pour syndromes coronariens aigus. Tunisie Medical.

